# Physiological and Clinical Researches on the Cerebro-Spinal Fluid

**Published:** 1842-10

**Authors:** 


### Art. XIII.

*Recherches Physiologiques et Cliniques sur le Liquide Céphalo-Rachidien ou Cerebro-Spinal.* Par F. Magendie.—*Paris*, 1842. 4to, pp. 164. With a Volume of Plates in Folio.

*Physiological and Clinical Researches on the Cerebro-Spinal Fluid.* By F. Magendie.—*Paris*, 1842.

In his preface to this essay, M. Magendie complains that justice has not been done to his former memoirs on this subject, and that the existence of the cerebro-spinal fluid, though mentioned by some writers, is passed over without any notice by most.

"This silence, however," says he, "does not surprise me, for the fluid to which I have given the name of *céphalo-rachidien* has always met with a singular fortune; everybody has seen it, it is impossible to open the skull without its flowing away under the eyes of the anatomist: it has served more than one as the basis of ingenious hypotheses, but nevertheless no one has yet devoted to it that serious attention which it deserves. Most medical men still regard it as a morbid product, while it is in reality one of the physiological conditions of life in man and in many animals.

"My intention, in choosing it as the subject of a special work, has been to place the fact of its normal existence beyond doubt, and to secure for it among the elements of the organism, that place which ought to be allotted to it." (Preface.)

M. Magendie appears to us to have greatly overstated the neglect with which his discovery has been treated. Before the publication indeed of his first paper in the *Journal de Physiologie* for 1825, the observations of Cotugno relative to the existence of a fluid after death in the cavities of the brain and spinal canal had been forgotten, and the fluid of the ventricles was generally regarded as the result of a morbid process. M. Magendie, to whom the investigations of Cotugno were at that time unknown, ascertained the normal existence of this fluid during life, and proved that it was not produced, as had been supposed, by the condensation of a vapour after death; he discovered its true situation to be between the arachnoid and pia mater, and satisfied himself of the existence of a distinct aperture of communication between the ventricles of the brain and the sub-arachnoid tissue of the spinal cord. The two former of these facts are now universally admitted, and the third is received by most anatomists, while all agree that for an acquaintance with them we are indebted to M. Magendie. The general adoption of his opinions, while no one sought to wrest from him the merit of having been the first to propound them, would have satisfied the craving for praise of most persons. Not so, however, with M. Magendie; from the date of his first communication in the Journal de Physiologie to the present time he has not ceased to reiterate his discovery; a great part of the first volume of his *Leçons sur les Fonctions et les Maladies du Système Nerveux*, published only three years ago, is occupied with the subject, and in the work now before us we have a *réchauffé* of former papers, without a single additional fact of importance. As, however, it is possible that many of our readers, though familiar with M. Magendie's results, are less perfectly acquainted with the grounds on which his conclusions rest, we have thought it desirable to prepare an analysis of the work before us; and to them we think the following pages will not be without interest.

M. Magendie's first proposition is that the brain does not completely fill the cranium, while the spinal cord is very far from occupying the whole of the vertebral canal. With reference to the spinal canal this fact is extremely obvious, as is shown in plate iii. figs. 4, 5, 6, and 7. In old persons, particularly in such as have sunk into a state of dementia, a similar condition of the brain is very evident, which, shrunk from its natural proportions, is far from filling up its bony case. If, bearing in mind the appearance presented in old persons, the anatomist examines the head of the adult or child, he will perceive that in them the brain by no means fills the cranium, but that there are between them many interspaces: or, if he should chance to examine a case in which this state of things does not exist, the condition of the brain will strike him at once as an exception to the general rule and the effect of some morbid process. He will find that the cerebral convolutions are flattened, that the sulci have disappeared, and the hypertrophied brain, instead of its peculiar undulating surface, will present a smoothness and a polish like that of a plaster cast, giving an exact copy of the interior of the skull.

These facts, and the frequency with which we read in accounts of post-mortem examinations, mention of the escape of fluid on opening the cranium, may be admitted as proofs that the brain and spinal cord do not completely fill the cavities which contain them. To prove the existence of a fluid in the cranium and spinal canal is the next step of the enquiry. Cotugno, though aware of the existence of such a fluid in the dead body, doubted whether it was present in the living subject, and the experiments on dogs which he instituted for the purpose of ascertaining this point, led him, though erroneously, to decide the question in the negative. M. Magendie observes that if the arches of the vertebræ of a living animal are removed, and the dura mater of the cord is exposed, the membrane will not collapse on the admission of air, but will continue to fill up the spinal canal. If now a puncture is made into the membrane, a jet of fluid will take place, and the dura mater will gradually wrinkle, collapse, and become closely applied to the spinal cord. The same phenomena may be noticed in examining a body immediately after death, but after the lapse of some hours, the greater part of the fluid becomes imbibed by the surrounding tissues, and the dura mater presents the same wrinkled appearance as it does when the fluid has been evacuated by puncture.

The existence of this fluid admitted, its seat is the next question which suggests itself. The supposition that it is contained in the cavity of the arachnoid, though very natural, is erroneous. It is found in two distinct localities: first, in the interspace between the arachnoid and pia mater; second, in the cavities of the cerebrum and cerebellum in the human subject, and likewise in those of the olfactory nerves, optic lobes, and spinal cord of some animals.

Closely as the arachnoid in many points resembles other serous membranes, it yet presents some peculiarities of arrangement which have been overlooked, owing to the little attention that has been paid to the existence of the cerebro-spinal fluid. Instead of adhering closely to the organ it invests, as the pericardium does to the heart, it is separated from the brain by a tolerably wide interspace, in which this fluid is situated. The whole spinal cord is thus bathed in the fluid, which forms a layer wider in some parts, narrower in others, according to the shape of the canal and the size of the cord in different places. One result of its presence, even in those situations where it is least abundant, is that the nerves float in it, and are thus kept separate from each other instead of being in close contact as they appear in the dead subject. In the skull the disposition of the fluid is similar, and the nerves are bathed in it to their exit from the cranium just as in the spinal canal. There are some situations, however, where it accumulates in such large quantity that they may with propriety be distinguished by the name of *confluences.*

"The first or posterior confluence, which is by far the largest, is situated below and behind the cerebellum. The second or inferior confluence is in front of the annular protuberance and between the crura cerebri, and lodges the basilar artery. The third or superior confluence is placed behind, above, and on the sides of the pineal gland. The fourth or anterior confluence exists above the decussation of the optic nerves, and below the pars cinereum which closes the third ventricle below and in front. Two other smaller confluences, lateral confluences, might be mentioned which bathe the ganglion of the fifth pair on each side." (p. 25.)

The same fluid which occupies the sub-arachnoid tissue penetrates, according to M. Magendie, into the ventricles. In the discovery that the fluid of the ventricles communicates with that in the spinal canal, the French physiologist was anticipated by Cotugno, but the question as to the way by which this communication is effected still remains undetermined. There is said to be an opening, called from its discoverer the *foramen of Bichat*, by which the arachnoid passes between the venæ Galeni, just where they enter into the straight sinus. The existence of this aperture is denied by Magendie, and in his scepticism on this point he is supported by many authorities, and by the researches of Martin St. Ange, which were specially directed to this subject. Some anatomists, however, whose opinion is entitled to great weight, admit the existence of the foramen of Bichat. Among their number is Arnold, whose *Annotationes de velamentis cerebri et medulla spinalis* deserve a different treatment than to be passed over in utter silence. But, even on the hypothesis that the foramen of Bichat is a natural and not an artificial opening, it would not at all answer the purpose of a means of communication between the ventricles and the subarachnoid tissue, since it leads, according, to Bichat, into the cavity of the arachnoid.

"But the real, constant, and normal opening, by which the cerebro-spinal fluid invariably passes in entering or in leaving the ventricles, is not at the place mentioned by the illustrious author of the *Anatomie générale;* it is to be found at the lower end of the fourth ventricle, at the spot called the *calamus scriptorius* by old anatomists. To convince one's self of the existence of this orifice, nothing more is necessary than to lift up and to separate slightly from each other the lobules of the inferior vermiform process of the cerebellum; when, without tearing any of the vascular adhesions, which connect this part of the cerebellum with the pia mater of the cord, the angular excavation which terminates the fourth ventricle is distinctly evident. Its surface is smooth and polished, and is continued into the ventricle of the cerebellum. Such is the anterior part of the opening: its lateral and superior parts are formed by the choroid plexuses of the organ, and by a lamina of medullary substance, the valve of Tarin, which varies much in size, and which is attached to the projecting part of the side of the fourth ventricle. As to the form and dimensions of the opening, they vary much in different subjects and according to the quantity of the cerebro-spinal fluid, and when this fluid is very abundant, the opening will admit the end of the finger. Usually, and when no more than the normal quantity of fluid is present, the aperture scarcely exceeds two or three lines in diameter in every direction. It is also frequently divided into several compartments by vessels which pass from the medulla oblongata to the cerebellum. Sometimes it is contracted by one or both of the posterior arteries of the cerebellum which pass across it."* (p. 28.)

On a subject still open to doubt, as is most certainly the existence of this aperture, which is not admitted by Arnold, some notice of the arguments against it might be looked for, but are not found in this essay of M. Magendie. They are, however, very fairly stated by M. Cruveilhier in his Anatomie Descriptive under the following heads:

1st. The form and disposition of the opening do not present any of the characters of a natural aperture, but it is irregular in shape, and its edges have a torn appearance, while a little triangular tongue of membrane may be found attached to the inferior vermiform process of a size and shape such as exactly to close the aperture. We may also add that the description given by Martin St. Ange tallies far less with the characters of a natural opening, than does that which we have quoted above from M. Magendie. St. Ange says expressly that it is "a mere cleft, by no means such an opening as had been asserted by some, and it is impossible to assign to it any dimensions, since sometimes there is no appearance of an opening. In short it is not a free and distinct canal; but vessels and cellular filaments pass in front of it."

2d. In the dog and sheep the fibrous lamina which forms the floor of the fourth ventricle is not perforated, and M. Cruveilhier has met with a similar arrangement five or six times in the human subject, in cases where not the slightest trace of any morbid process was to be found either in the membranes or substance of the brain or spinal cord.

3d. In many cases of chronic hydrocephalus, the ventricles contained several pounds of fluid, while none whatever was present in the sub-arachnoid cellular tissue.

4th. In the brain of many children who have died with all the symptoms of acute hydrocephalus, M. Cruveilhier has found the lateral ventricles extremely capacious but empty, and the question has suggested itself to him whether in these instances the rupture of the membrane has not given passage to the fluid, while in ordinary cases it prevents its escape.

Notwithstanding these facts, M. Cruveilhier is induced to admit the normal existence of this aperture: 1st. From finding it, in the immense majority of cases both in the fœtus and in the adult, even when the brain has been removed with the greatest care. 2d. From the fact that in apoplexy of the ventricles bloody serum is always met with in the sub-arachnoid cellular tissue. 3d. From observing that if a coloured fluid is injected into the ventricles of the brain it always penetrates into the sub-arachnoid cellular tissue, and vice versâ.

This last occurrence might, we think, be adequately explained by endosmosis, and Martin St. Ange's investigations do not by any means

* Reference is made to drawings of the opening, but none have been published in the volume of plates.

clearly establish the existence of a distinct opening. He suspended bodies by the head or by the heels, and found that the fluid of the ventricles passed into the sub-arachnoid tissue, and that of the sub-arachnoid tissue into the ventricles, according to the position of the body. This did not, however, take place immediately, as it might be expected to do if it flowed through a distinct opening, but he says " *Cette transmission de liquide se faisait il est vrai, avec plus ou moins de facilité, et quelquefois même avec beaucoup de lenteur.*"*

In his estimate of the quantity of this fluid, M. Magendie agrees pretty closely with Cotugno. It varies according to the age and size of the patient, and usually bears an inverse proportion to the volume of the encephalon. It seldom, however, is less than two ounces, and often amounts to five ounces. M. Magendie regards the pia mater as the source of the secretion, which may be seen transuding from the membrane if it is exposed in a living animal. One striking point connected with its secretion is the rapidity with which, like the aqueous and vitreous humours, it is reproduced after having been evacuated. Within twenty-four hours after its removal, by puncture of the spinal canal, it will be found to have reaccumulated in as large a quantity as before, and this experiment may be repeated with the same result on several successive days.

The cerebro-spinal fluid is subject to movements synchronous with those of respiration, in which it advances towards the frontal extremity of the ventricles and then retires; but M. Magendie has never observed the fluid of the ventricles communicate in animals with that of the spinal canal. In the human subject, however, he does not doubt but this communication takes place, the opening of the fourth ventricle being so much more free in man than in animals, and in proof of this he appeals to the manifest distension of the brain produced by compressing the sac of a spina bifida. These movements are owing to the same causes as those of the brain, and like them, though very evident when the parietes of the cranium are removed, and the air exerts direct pressure on the organ, they are doubtless much less marked when the skull is entire.

The result of chemical analysis establishes, in M. Magendie's opinion, the fact that this fluid is of a peculiar nature, and that it differs essentially from mere serum. M. Lassaigne and M. Haldat found in it osmazome, albumen, various salts, and about 97 per cent. of water. M. Conerbe states that it contains the following elements, but has not mentioned the proportions in which they exist in it:

" 1. Animal matter, insoluble in alchohol and ether—soluble in alkalis: it is analagous to the neurilema of the brain. 2. Albumen. 3. Cholesterine. 4. Cérébrote.† 5. Chloride of soda. 6. Phosphate of lime. 7. Salts of potash. 8. Salts of magnesia." (p. 50.)

M. Magendie regards the uses of the fluid as almost exclusively mechanical. It occupies all the irregularities of surface presented by the brain and spinal cord, it distends the membranes and transmits to the whole extent of the cranio-spinal parietes pressure made upon one part, and both from within and from without it presses on the encephalon. In other words, its uses are such as result from the known properties of

* Journal Hebdomadaire, Janvier, 1830, p. 104.

† The name applied by M. Conerbe to the white fatty matter of Vauquelin.

fluids. To these facts, which are stated with much unnecessary expenditure of words, we have no objection to make. But M. Magendie observes:

"The consequences of this pressure influence the general conformation of the head, and doubtless also of the spinal column from the earliest period of embryonic and fœtal existence, during which the parietes of the cranium and vertebral column are not yet solid. To what other cause are we to refer the spherical form of the head at a time when the brain is not yet in existence? The cerebro-spinal fluid protects the formation of the cerebro-spinal nervous axis, just as the fluids of the ovum, and especially the liquor amnii, defend and protect the whole fœtus in the cavity of the uterus. There must even exist a kind of antagonism between the cerebro-spinal fluid, which tends to distend the parieties of the cranium and the spine, and the fluid of the amnios, which compresses them on every side, and tends to make them assume the smallest possible volume." (p. 54.)

M. Magendie stated in a former part of this essay, that the fluid is secreted by the pia mater, but no trace of the membranous envelopes of the brain was found by Tiedemann before the eighth week, and the pia mater is not distinguished till even a later period. The earliest form which the brain presents is that of a number of vesicles filled with fluid, which gradually diminishes in quantity as the walls of the vesicles thicken, and at length entirely disappears in some parts, owing to the deposition of solid matter. Some relation may probably exist between the fluid of the cerebral vesicles and the sub-arachnoid fluid of after life. It would be interesting to ascertain the exact nature of this relation, and the solution of this question would necessarily throw light upon other still debateable subjects, and especially on that of the lining membrane of the ventricles—a point which Arnold has treated with less than his accustomed clearness. These are questions, however, the elucidation of which calls for patient and laborious investigation. Statements so loose and incorrect as those we have just quoted can advance neither the interests of science nor the reputation of their author.

The second part of the work treats of the cerebro-spinal fluid in disease, which M. Magendie refers to the two heads of alterations in quantity, or changes in character.

The quantity of the fluid may either be increased or diminished, and M. Magendie remarks, that "When once we become aware of the existence of the cerebro-spinal fluid the thought naturally suggests itself, that all accumulations of fluid in the interior of the cranium or spinal canal are merely accidental or morbid modifications of this fluid—a fact which it will be easy enough to establish." (p. 66.)

It has doubtless long before this struck our readers, that one question most intimately connected with the subject of this essay has been passed over by the author in utter silence. We allude to the nature and quantity of the secretion which takes place into the sac of the arachnoid—the differences between it and the cerebro-spinal fluid and the influences of disease upon each. Now, no person previously unacquainted with the subject could imagine, after a perusal of this work, that any secretion is ever formed in the sac of the arachnoid. But instead of investigating this matter, M. Magendie makes the broad assertion, that all accumulations of fluid within the cranium are produced by the liquo cerebo-spinalis, though there are cases of chronic hydrocephalus in abundance in which the sac of the arachnoid has been found distended by fluid; and the plates of M. Cruveilhier furnish instances in which atrophy of the brain has coexisted with accumulation of fluid in the cavity of the arachnoid, as well as others in which this condition was associated with increase of the liquor cerebro-spinalis. There is, moreover, no invariable connexion between the accumulation of fluid in the ventricles and effusion into the sub-arachnoid tissue, as M. Cruveilhier candidly admits, though no allusion to such a fact is to be found in the pages of this essay. The observations of M. Cruveilhier refer only to cases of chronic hydrocephalus; but the same fact may be observed in the bodies of patients who have died of acute hydrocephalus—fluid being found in the ventricles when none is contained in the sub-arachnoid tissue, and the sub-arachnoid fluid being sometimes abundant in cases where the ventricles are found to be empty.

The obliteration of the aperture at the calamus scriptorius is mentioned by M. Magendie as a probable cause of the accumulation of a morbid quantity of this fluid in cases where it has been found confined to the ventricles. But after M. Cruveilhier's statement, that he has found this aperture obliterated without any morbid condition of the brain or spinal cord, we feel rather sceptical on the point. The influence of tubercles or other tumours of the brain in causing an accumulation of fluid in the ventricles is a fact with which the profession has long been familiar. The quantity of the cerebro-spinal fluid has been found increased in cases where some obstacle has existed to the venous circulation in the brain, or where there has been habitual congestion of the organ; also in instances where the brain is either partially or generally diminished in size. Under opposite circumstances it is found in less quantity than natural, as when the brain is hypertrophied or the cranial bones have acquired a preternatural thickness.

The diminution in the quantity of this fluid usually takes place slowly and by degrees: hence its symptoms are, for the most part, very obscure, and marked by the graver cerebral disease of which it is merely a consequence. In many cases, too, the increase in the fluid is gradual, and months or even years elapse before it attains its maximum. Neither are the cerebral functions, nor is the power of motion seriously impaired; or if such disturbance should take place, other lesions of the brain will be found to which they may be attributed. Instances, however, do occur in which a sudden accumulation of the fluid takes place, when symptoms of what has been called serous apoplexy take place, which closely resemble those produced by the injection of a large quantity of liquid into the cranium or spinal canal of animals.

Besides the increase or diminution in the quantity of the cerebro-spinal fluid, it is liable to modifications of its physical qualities owing to the admixture of blood or pus, or of cerebral matter, as is sometimes the case in softening of the brain. Of these changes that produced by the admixture of blood seems the most important; and the invariable admixture of blood with the sub-arachnoid fluid in cases where hemorrhage has taken place into the ventricles appears to be one of the strongest arguments in favour of a communication between the interior of the brain and the sub-arachnoid tissue.

The symptoms which characterize this occurrence are not very easy to interpret, since in these cases there exist at the same time laceration and compression of the brain, coagula of blood, and blood mingled with the fluid. From a comparison of a good many cases, M. Magendie concludes that—

"1. When a partial admixture has taken place at the surface of the cerebro-spinal organ, as when the extravasated matter has completely assumed the place of the fluid, phenomena of compression exist in direct relation to the quantity of the effusion and the extent of surface compressed.

"2. When a partial admixture takes place in the ventricles, somnolence and sometimes profound sleep are observed—phenomena which likewise exist in cases of simple accumulation of fluid.

"3. Lastly, when this mixture is general, the same accidents occur, but they supervene more rapidly, like the cause which produced them, and there always exist various signs of compression, according to the quantity of the foreign matter, the points where it collects, and especially according to the greater or less rapidity of its effusion." (pp. 122-3.)

The work concludes with some interesting though confessedly incomplete observations on the cerebro-spinal fluid in the different classes of vertebrate animals. It exists in greatest abundance in the higher orders of animals, among whom the movements of the nervous system are most active, and diminishing gradually among the lower classes of animals, it finally disappears entirely in some species of fish. But, independently of its relation to the size of the nervous centres, its quantity is greatly influenced by the different modifications in the mechanism of the protecting envelopes and of the circulating system of the brain and spinal cord.

In man and the higher animals there exist an external sub-arachnoid and an internal, intra-ventricular fluid. The quantity of the former is in general greater in proportion to the fragility of the cranium, and in aged persons whose bones are very brittle this external fluid is usually very abundant. When some peculiar structural arrangement of the bones of the cranial arch provides the brain with another protection, the layer of fluid diminishes. This modification is already found in some ruminants; it is at its maximum in birds, reptiles, and fishes, in which there exist mere traces of the sub-arachnoid cerebral fluid.

The spinal cord is protected in the same manner as the brain by this fluid, which is especially abundant in situations such as the neck, where numerous and extensive movements take place. It is not, however, merely as a means of protection to the cerebro-spinal axis that this fluid deserves attention; but it fulfils an important office with reference to the circulation in those parts. All the vessels which supply the brain and spinal cord run beneath the arachnoid membrane; and we find the chief accumulations, the confluences of the sub-arachnoid fluid in the cranium around the largest vesicular trunks. Hence it follows that the circulation is carried on without interruption in the cerebral vessels, owing to the presence of the cerebro-spinal fluid, which prevents the brain from compressing them, as it must necessarily do if it exactly filled the whole of the cavity of the cranium. This fluid, moreover, by occupying the interspaces between the convolutions into which the pia mater dips, constantly keeps the two surfaces apart, and thus favours the circulation in the capillaries. In proof of this may be adduced the fact that in proportion as the cerebral convolutions disappear in different tribes of animals the fluid on the surface of the brain diminishes in quantity; and in birds and reptiles, in which no convolutions exist, it has entirely left the surface of the organ to collect at the confluences which surround the large vessels. The spinal cord is surrounded by a layer of fluid wider in proportion than that which surrounds the brain—a provision called for by the enormous size which the vertebral sinuses are capable of attaining; since, unlike the sinuses of the brain, they are not contained in unyielding fibrous canals.

The various classes of animals exhibit another use of this fluid—namely, that of protecting the nerves from pressure up to the point of their exit from the cranium and vertebral canal. This use of it is illustrated by the fact, that while in the human subject the Gasserian ganglion is surrounded by a confluence of this fluid, there are some animals—as the horse and ox—in which it does not exist. In them, however, that portion of the dura mater which attaches the ganglion to the petrous portion of the temporal bone is ossified, and the ganglion is thus placed secure from pressure external to the cranial cavity.

Some historical notices, by a pupil of M. Magendie, of the opinions of the older anatomists concerning the fluid in the cranium, are given as an appendix to the work. The observations which we have just noticed on the cerebro-spinal fluid in animals form its only pretensions to novelty: all the other facts and opinions it contains will be found in the Journal de Physiologie, with the great advantage of not being expanded by a tedious verbosity to a bulk sufficient to occupy a quarto volume. Still the subject is one of interest and importance, and we are glad to have had the opportunity of placing a complete view of it before our readers. The consideration of it may be useful, both in a pathological and practical point of view; and to that numerous class of juvenile and senile theorists whose scanty philosophy is in the direct ratio of their knowledge, and whose feeble judgment is in the inverse ratio of their imagination, the facts and views we have recorded may prove a mine of boundless wealth.

---

